# Multi-moded high-index contrast optical waveguide for super-contrast high-resolution label-free microscopy

**DOI:** 10.1515/nanoph-2022-0100

**Published:** 2022-06-20

**Authors:** Nikhil Jayakumar, Firehun T. Dullo, Vishesh Dubey, Azeem Ahmad, Florian Ströhl, Jennifer Cauzzo, Eduarda Mazagao Guerreiro, Omri Snir, Natasa Skalko-Basnet, Krishna Agarwal, Balpreet Singh Ahluwalia

**Affiliations:** Department of Physics and Technology, UiT The Arctic University of Norway, Tromsø 9037, Norway; Department of Microsystems and Nanotechnology, SINTEF Digital, Gaustadalleen 23C, 0373 Oslo, Norway; Department of Pharmacy, Faculty of Health Sciences, UiT The Arctic University of Norway, Tromsø 9037, Norway; Department of Clinical Medicine, UiT The Arctic University of Norway, Tromsø 9037, Norway; Department of Clinical Science, Intervention and Technology, Karolinska Insitute, 17177 Stockholm, Sweden

**Keywords:** coherence of light, high throughput imaging, high-contrast label-free imaging of nano carriers and biological cells, intensity fluctuation algorithms, label-free microscopy, multi-moded high-index contrast waveguide

## Abstract

The article elucidates the physical mechanism behind the generation of superior-contrast and high-resolution label-free images using an optical waveguide. Imaging is realized by employing a high index contrast multi-moded waveguide as a partially coherent light source. The modes provide near-field illumination of unlabeled samples, thereby repositioning the higher spatial frequencies of the sample into the far-field. These modes coherently scatter off the sample with different phases and are engineered to have random spatial distributions within the integration time of the camera. This mitigates the coherent speckle noise and enhances the contrast (2–10) × as opposed to other imaging techniques. Besides, the coherent scattering of the different modes gives rise to fluctuations in intensity. The technique demonstrated here is named chip-based Evanescent Light Scattering (cELS). The concepts introduced through this work are described mathematically and the high-contrast image generation process using a multi-moded waveguide as the light source is explained. The article then explores the feasibility of utilizing fluctuations in the captured images along with fluorescence-based techniques, like intensity-fluctuation algorithms, to mitigate poor-contrast and diffraction-limited resolution in the coherent imaging regime. Furthermore, a straight waveguide is demonstrated to have limited angular diversity between its multiple modes and therefore, for isotropic sample illumination, a multiple-arms waveguide geometry is used. The concepts introduced are validated experimentally via high-contrast label-free imaging of weakly scattering nanosized specimens such as extra-cellular vesicles (EVs), liposomes, nanobeads and biological cells such as fixed and live HeLa cells.

## 1 Introduction

Label-free microscopy circumvents the need for exogenous contrast agents. However, this gives rise to challenges such as poor contrast and low resolution while performing far-field label-free microscopy of weakly scattering biological specimens. Diffraction-limited resolution arises due to the inability to capture high spatial frequencies of the specimen in the far-field, whereas poor contrast in the optical regime is attributed to a weak scattering signal in comparison to the illuminating light. Moreover, illuminating these samples with a highly coherent light source like a laser can lead to speckle formation, degrading the image quality [1]. Hence, the key idea in this paper is to describe mathematically the physical mechanism and demonstrate experimentally how a high-index contrast multi-moded optical waveguide helps mitigate the abovementioned challenges in label-free microscopy.

High-contrast label-free images of weakly scattering specimens typically use holographic interferometric setups [2–4], holographic noninterferometric setups [5, 6], sequential illumination of the sample and iterative stitching in Fourier space [7, 8], multiple 2D holographic measurements for 3D reconstruction of refractive index of the sample via inverse scattering [9], multiple intensity-only measurements for tomographic reconstruction [10, 11], a physical stop to block background light [12], phase-rings [13] etc. Most of these techniques illuminate the entire volume of the sample, require multiple frames for reconstructing the final image or/and typically use incoherent white light or LED [14] as the light source which has lower photon degeneracy [2]. On the other hand, sources with higher photon degeneracy like lasers can generate coherent artifacts. This problem can be mitigated via optical sectioning of the sample, as in total internal reflection microscopy [15]. A typical way of generating an evanescent wave illumination is using a total internal reflection fluorescence (TIRF) objective [16]. Rotating Coherent Scattering microscopy (ROCS) [17] uses an evanescent field generated by a diode laser passed through a rotating diffuser to illuminate the sample from all azimuthal directions. However, a high magnification/numerical aperture (N.A.) TIR objective lens (e.g., 60–100× > 1.33 N.A.) is typically used in ROCS, thus limiting the field-of-view (FoV). Another approach to generating evanescent fields and over larger areas is via an optical waveguide, i.e., a photonic chip [18–26]. Previous chip-based label-free microscopy works use an incoherent white light source [23], fluorescent nanowire ring illumination [24], index-matched waveguide geometry [25], polymer fluorescent films [26], Fourier Ptychography via single-mode waveguide [27] etc. to suppress stray light that is detrimental while imaging weakly scattering specimens. However, in this article, a high index contrast optical waveguide guiding laser along its length is demonstrated as a feasible secondary light source for superior contrast and high-resolution imaging of weakly scattering specimens. As opposed to other label-free waveguide techniques which require multiple images with complicated optical setup [27], incoherent light source or/and in combination with index-matched waveguides [23–26], here in this work a high-index contrast multi-moded waveguide guiding a coherent laser light is engineered as a partially coherent secondary light source for single-shot imaging with superior contrast.

**Figure 1:**
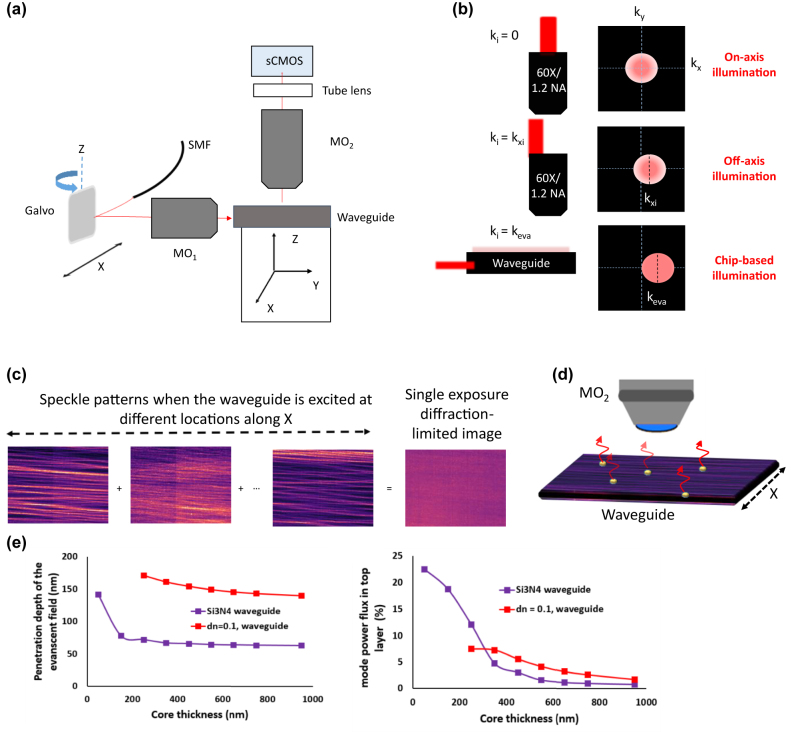
Basic concepts of cELS. (a) Schematic of cELS experimental setup. (b) Influence of obliquity of illumination in Fourier domain (*k*_*x*_–*k*_*y*_ domain). Three different cases corresponding to on-axis epi-illumination (*k*_*i*_ = 0), off-axis epi-illumination (*k*_*i*_ = *k*_*xi*_) and waveguide chip-based illumination (*k*_*i*_ = *k*_eva_) and the corresponding object spectrum they sample are shown. Flat-field like illumination provided by high-refractive index chip provides access to the higher sample spatial frequencies. (c) The addition of multiple speckle patterns at the camera plane helps suppress the speckle noise. (d) Schematic representation of an optical waveguide supporting several guided modes and some scattering objects that convert the evanescent waves into scattering waves is also given. (e) The penetration depth and field intensity of a TE1 mode of Si_3_N_4_ high index core (Δ*n* ≈ 0.5) waveguide and an index matched waveguide (Δ*n* ≈ 0.1) are provided here.

Through this work, which uses the experimental setup shown in Figure 1(a), the following concepts are proposed: (1) Multi-moded optical waveguide as a partially coherent light source, (2) physical mechanism of high-contrast label-free image formation using a multi-moded waveguide, (3) feasibility of employing intensity fluctuation algorithms [28], typically used in fluorescence microscopy, to utilize fluctuations in intensity induced by the multiple modes [29] coherently scattering off the sample and (4) for isotropic sample illumination, a four-arm crossing waveguide is used to mitigate the challenge of limited angular diversity between the modes of a straight waveguide. An overview of waveguides, modes and fabrication of the chips is given in Supplementary sections S1-S4 and Figures S(1)–S(4). A comparison between the different chip-based label-free works is provided in Table 1 of the supplementary material. In addition, this approach based on photonic chips offers several advantages: (1) the decoupled illumination and detection scheme allows only the scattered light off the sample to reach the camera. A comparison between the different illumination schemes is given in Figure S5 of the supplementary material. (2) The use of high refractive index waveguide material (*n* ≈ 2) enables accessing higher spatial frequencies of the sample [30], see Figure 1(b), that are typically inaccessible using conventional free-space bulk optics approach or using index-matched waveguide geometries [25]. The high index core, *n*_eff_ = 2, reduces the speckle size that can be formed to about 2 ***π***/(*k*_e_ + *k*_out_), where *k*_e_ = 2 ***π***.*n*_eff_/*λ*_vac_, *k*_out_ = 2 ***π***.N.A./*λ*_vac_, *n*_eff_ is the effective index of mode, *k*_e_ is the magnitude of the incident evanescent wave vector, *k*_out_ defines the passband of the microscope and *λ*_vac_ is the vacuum wavelength [31]. (3) The addition of multiple modes within the integration time of the camera helps suppress speckle noise as shown in Figure 1(c). (4) Any perturbation in the index at the core-cladding interface scatters light into the microscope objective (MO) as shown in Figure 1(d). (5) The use of a coherent light source like a laser helps focus very high-power into thin waveguide geometries. The lack of specificity in label-free imaging and consequently multiple scattering issues are mitigated by the evanescent field excitation of the low-loss high refractive index material thin (150 nm) waveguides. This provides excellent optical sectioning to about less than 100 nm and high field intensities [32], as shown in Figure 1(e). As opposed to index-matched optical waveguides [25], a thin (150 nm) high-refractive index contrast Si_3_N_4_ waveguide as used in this work significantly enhances the intensity in the evanescent field, with up to 10–15% of the mode power flux present in the evanescent field, Figure 1(e). A high-index waveguide thus enables both the collection of higher spatial frequencies and generates high field intensity that are crucial while imaging nano-sized weakly scattering objects. This work utilizing the coherent scattering of the multiple modes of the waveguide to mitigate the coherent speckle noise is referred to as chip-based evanescent light scattering (cELS).

## 2 Optical setup and imaging conditions

The schematic of cELS experimental setup is shown in Figure 1(a). The coherent laser (Cobolt Flamenco 660) light, *λ*_vac_ = 660 nm, is coupled into a single mode fiber that delivers collimated light via a collimator onto a galvo mirror which is free to rotate along the *z*-axis. The galvo helps steer this light onto the back focal plane of a microscope objective (Olympus LMPanFL N 50×/0.5 NA), MO_1_. MO_1_ focuses the incident collimated light onto the input facet of a waveguide. This configuration enables scanning of the incident light on the input facet of the waveguide, exciting different sets of guided modes for each incident location. The waveguide is mounted on a high-precision piezo electric XYZ-translation stage. The sample to be imaged is placed on top of the waveguide core. The evanescent light that interacts with the sample, gets scattered and is collected by a microscope objective MO_2_. Via a 4*f* setup, the scattered light is imaged onto an sCMOS camera (Hamamatsu C13440-20CU). The exposure time of the camera for the different experiments presented in this article is typically about 30 ms. The galvo oscillation rate is set at 1013 Hz for the waveguide widths used in this experiment, a prime number, which causes a spatial redistribution of the excited modes within the exposure time of the camera. Throughout this article, experiments have been carried out using a silicon nitride (Si_3_N_4_) waveguide. The fabrication of Si_3_N_4_ waveguide and the preparation, characterization, and labeling strategies of biological samples such as liposomes, EVs and cells are given in Sections S4–S7 of the supplementary material.

## 3 Theory of image formation in cELS

The theory section provided here and in the Supplementary Material focuses on mainly three concepts: (1) transmitting near-field information to far-field, (2) waveguide as a partially coherent light source and (3) multiple modes induce fluctuations in intensity that aid in the generation of high-contrast images.

### 3.1 Near-field information to far-field

If an ideal waveguide without any sample is imaged, no light will reach the camera plane. However, any perturbation in the refractive index at the core-cladding interface can scatter photons into the camera [28]. The physical mechanism behind the conversion of nonpropagating evanescent waves into propagating waves may be understood from the following simplified illustration [31, 33]. A two-dimensional sample is illuminated by an incident field $E\left(x,y,z\right)$ . Let us represent the two-dimensional Fourier transform of this field by $\tilde{E}\left(\alpha ,ß;z\right)$ where *α*, *ß* and *γ* are spatial frequencies with respect to *x*, *y* and *z* axis respectively, i.e., propagation vector $\vec{k}=\alpha \hat{x}+ß\hat{y}+\gamma \hat{z}$ . The magnitude of the wave vector of a waveguide mode is $\frac{2\pi }{\lambda_{\text{vac}}}n_{\text{eff}}$ , where *n*_eff_ is the effective mode index. The evanescent wave vector corresponds to the largest spatial frequency components of the field. This field interacts with a thin sample placed at *z* = 0. The sample may be represented by a transmission function *T*(*x*, *y*). Invoking the Born approximation, just after the thin sample the field becomes [31]

(1) $$E_{\text{sample}}\left(x,y;0\right)=T\left(x,y\right)E\left(x,y;0\right)$$

By the property of Fourier transform, Eq. (1) may be represented alternatively as the convolution of the two signals in the spatial frequency domain as

(2) $${\tilde{E}}_{\text{sample}}\left(\alpha^{\prime },{ß}^{\prime };0\right)=\overset{\infty }{\underset{-\infty }{\iint }}\tilde{E}\left(\alpha ,ß;0\right)\hat{T}\left(\alpha -\alpha^{\prime },ß-{ß}^{\prime }\right)d\alpha dß$$

But the illuminating field may be represented via the sifting property of the delta function as follows

(3) $$\tilde{E}\left(\alpha ,ß;0\right)=\overset{\infty }{\underset{-\infty }{\iint }}\tilde{E}\left(\tilde{\alpha },\tilde{ß};0\right)\delta \left(\tilde{\alpha }-\alpha ,\tilde{ß}-ß\right)d\tilde{\alpha }d\tilde{ß}$$

Combining Eqs. (2) and (3) the spatial frequencies of the sample represented by $\hat{T}\left(\alpha ,ß\right)$ gets convolved with the spatial frequencies of the incident field. Or in other words, if a mode of an evanescent field is represented by $\delta \left(\alpha_{\text{eva}},\;{ß}_{\text{eva}}\right)$ , then the electric field just after the thin sample contains the shifted object spectrum $\hat{T}\left(\alpha -\alpha_{\text{eva}},ß-{ß}_{\text{eva}}\right)$ , where *α*_eva_ and *ß*_eva_ are the spatial frequencies of the evanescent wave illuminating the sample. This is illustrated in Figure 1(b). If the shifted version of the function falls within the passband of the microscope, those high spatial frequencies of the object will reach the camera plane. Thus, sub-diffraction limit sized features are captured using cELS due to the high *n*_eff_ of the waveguide core that is typically not accessible with conventional objective based illumination schemes or index-matched waveguide geometries.

### 3.2 Waveguide as a partially coherent light source

In conventional bright field imaging, the incident light $E_{\text{i}}\left(r,t\right)$ and the light scattered off the sample $E_{\text{s}}\left(r,t\right)$ reach the camera. The total complex scalar field at the camera plane is $E_{\text{T}}\left(r,t\right)=E_{\text{i}}\left(r,t\right)+E_{\text{s}}\left(r,t\right)$ . For weakly scattering specimens, the only modulation in the total field will be in its phase. But the intensity registered by the camera, $I\left(r,t\right)$ , will have no phase information and hence poor contrast, where ⟨⟩ represents time averaging. But while imaging using waveguides, only scattered light off the sample gets detected, i.e., $E_{\text{T}}\left(r,t\right)=E_{\text{s}}\left(r,t\right)$ . Therefore, the point will be visible with enhanced contrast as a bright spot on a dark background.

Consider two-point scatterers represented by *j* = 1, 2. The incident field induces Rayleigh dipoles [34] which radiate into the far-field. Let the field emitted by each emitter at the camera plane be given by $E_{j}\left(r,t\right)=E_{0}\left(r\right)\exp\left[\text{i}\varphi_{j}\left(r,t\right)-\text{i}\omega t\right]$ . Here we assume that the scatterers are identical, i.e., the radiated fields have the same amplitude. The total field intensity averaged over the integration time of the camera is then represented as [35]

(4A) $$I\left(r,t\right)=2{\left|E_{0}\left(r\right)\right|}^{2}+2{\left|E_{0}\left(r\right)\right|}^{2}〈\cos\left(\chi \left(r,t\right)\right)〉$$

where

(4B) $$\chi \left(r,t\right)=\varphi_{1}\left(r,t\right)-\varphi_{2}\left(r,t\right)$$

First, let us consider the situation of coherent illumination. Although the phases $\varphi_{1}\left(r,t\right)$ and $\varphi_{2}\left(r,t\right)$ are a function of time, the phase difference $\chi \left(r,t\right)$ is time-invariant and can be simply represented as $\chi \left(r\right)$ . The cosine term in Eq. (4A) becomes time invariant and therefore, the interference phenomenon is observed. On the contrary, in the case of incoherent illumination as in fluorescence imaging, the phase difference $\chi \left(r,t\right)$ is not time-invariant and the cosine term is a function of temporal variations. Since the phase fluctuations occur on a time scale much smaller than the integration time of the camera, the time-averaged cosine term tends to zero and therefore no interference is observed.

However, multi-moded illumination patterns inside the photonic waveguides presents a very interesting case. Let the electric fields emitted from the scatterers due to a particular mode “*m*” be represented as $E_{j,m}\left(r,t\right)=E_{0,m}\left(r\right)\exp\left[\text{i}\varphi_{j,m}\left(r,t\right)-\text{i}\omega_{m}t\right]$ , where the subscript *m* denotes the mode. Correspondingly, the subscript *m* may be introduced in *I*_*m*_(***r***, *t*) and $\chi_{m}\left(r\right)$ as well. Since the modes are coherent individually and with respect to each other, the time term in the function $\chi_{m}\left(r,t\right)$ is absent. At any given point in time *t*, due to galvo scanning, the mode combinations will be different. Representing the complex mode coefficients at a given time *t* as *a*_*m*_(*t*), the average intensity within a camera integration time due to all the mode combinations is given as

(5) $$\begin{array}{ll} I\left(r,t\right) & =\left\langle 2{\left|a_{m}\left(t\right)\right|}^{2}{\left|E_{0,m}\left(r\right)\right|}^{2}\right.\\ & \;\left.+2{\left|a_{m}\left(t\right)\right|}^{2}{\left|E_{0,m}\left(r\right)\right|}^{2}\cos\left(\chi_{m}\left(r\right)-\alpha_{m}\left(t\right)\right)\right\rangle \end{array}$$

where $\alpha_{m}\left(t\right)$ represents the phase of *a*_*m*_(*t*). The presence of a time-varying cosine term $\cos\left(\alpha_{m}\left(t\right)\right)$ which changes continuously with the galvo scan position implies that the average intensity shown in Eq. (5) is no longer coherent. However, it is also not strictly incoherent because the galvo scan times are comparable to the camera exposure time. To ensure that there is no strict correlation between the images acquired across different frames we set the galvo scan rate to a prime number. In essence, we realize a partially coherent illumination case per frame. Equation (5) may be understood as many speckle patterns getting added at the camera plane. As per the central limit theorem, the contrast of these speckles scales as $1/\sqrt{N}$ when added on an intensity basis, where *N* is the number of independent speckle patterns added [36]. This is illustrated schematically in Figure 1(c). In the experiments described here, the galvo oscillation rate is set at a prime number of 1013 Hz. The logic behind choosing a prime number can be understood as follows. During oscillation at each position of the galvo, a set of modes are excited in the waveguide that get coherently scattered off the sample onto the camera. Within one exposure time of the camera, the galvo would have oscillated (0.030 s × 1013 Hz ≈ 31) times and excited the modes. Due to a prime number setting, it will ensure that 31 distinct set of mode patterns or speckle patterns get averaged within the integration time of the camera. This condition will help suppress the speckle noise according to the Central Limit Theorem. Thus, the issue of coherent noise is mitigated via the usage of a multi-moded waveguide and galvo scanning, demonstrating a multi-moded waveguide as a partially coherent light source that enables high-contrast imaging.

### 3.3 Coherent scattering of modes enable super-contrast label-free imaging

**Figure 2:**
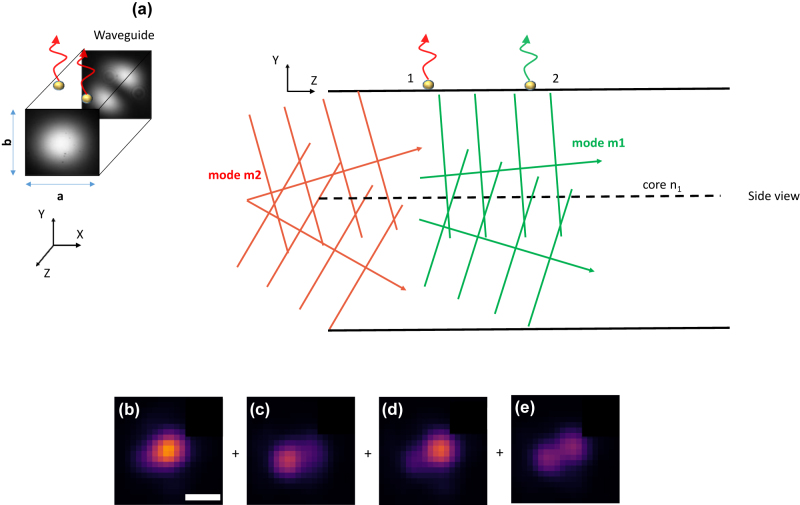
Theory of cELS image formation. (a) A rectangular waveguide with transverse widths “*a*” and “*b*” units guiding power via fundamental and higher-order modes is shown. The two modes m1 and m2 are shown in green and red color respectively in the side view diagram. The modes are decomposed into a pair of plane waves propagating at discrete angles with respect to the optical axis along *z*. The evanescent tails of these guided modes polarize particles 1 and 2 placed on the surface and scatter into the far-field. (b–e) Experimental demonstration of the theory of image formation using a multi-moded waveguide. The images are of 100 nm gold nanoparticles imaged using a 10X/0.25 NA MO. The multi moded speckle pattern causes variations in the intensity of the coherently scattered light. Scale bar 2 μm.

Consider two particles “1” and “2” placed on top of the waveguide surface as shown in Figure 2(a). The input coherent laser light excites a few modes of the waveguide. These modes are described mathematically as given by Eq. (S3) in the supplementary material. So, any mode “*m*” may be decomposed into a pair of plane waves propagating at angles ±*θ*_*m*_ with respect to the propagation direction, *z*-axis. For brevity only two such modes are shown in green and red in Figure 2. The tails of these modes extend into the cladding and polarize the particles “1” and “2”. The particles then radiate into the far-field as described earlier. As per the first order Born approximation, the incident and scattered waves can be assumed to have the same phase [34]. Therefore, the phase difference between the scattered waves off the two particles will be dependent only on the positions of these particles on the waveguide. Therefore, the imaging process may be described as follows.

Two particles separated by one Rayleigh distance = 0.61*λ*/NA, are located on a rectangular waveguide with transverse widths “*a*” and “*b*” units as shown in Figure 2. For *λ* = 660 nm and NA = 1.2., the two particles can then be assumed to be located at points with coordinates (0, *b*/2, 0) and (0.508*a*, *b*/2, 0). In the case of incoherent imaging, i.e., if particles “1” and “2” are fluorescent beads, they will be just resolved in an ideal microscope as per Rayleigh’s resolution criteria. However, the coherent scattering off the particles by the multi-moded waveguide chip presents the following interesting scenarios in contrast the incoherent fluorescent imaging. This is listed below.

1. The two particles are illuminated by the same mode. For example, consider illumination with TE_11_ mode (*m* = 1, *n* = 1) which is described in Eq. (S3) of the supplementary text. Substituting the above-mentioned particle’s location into Eq. (S3), the phase difference between the coherently scattered light reaching the detector will be approximately 1.6 radians. Due to interference between these coherently scattered fields as described by Eq. (4A), the particles will no longer be resolved as per Rayleigh’s criteria. This contrasts with these two particles getting resolved as in the case of incoherent imaging.
2. The two particles are illuminated by say TE_11_ (*m* = 1, *n* = 1) and TE_21_ (*m* = 2, *n* = 1) mode. Substituting the abovementioned particle locations into Eq. (S3), the phase difference between the fields is seen to be ***π*** radians. As a result, the particles get resolved in label-free mode as per Rayleigh’s criteria.

Now as the galvo oscillates to vary the illumination patterns in the waveguide, the complex mode coefficient *a*_*m*_(*t*) changes with time, hence, both the amplitude and phase of the scattered light change. An image stack so acquired over time exhibits fluctuations in intensity. This is shown via the experimental results in Figure 2(b). As a result, image contrast can be enhanced by performing the average or standard deviation of such an image stack.

Naturally, the feasibility of employing intensity-fluctuation based algorithms to such an image stack exhibiting fluctuations in intensity is worth analyzing. These fluctuation-based algorithms are typically used in fluorescence microscopy to circumvent the diffraction limit. However, applying these fluorescence-based algorithms in the partial coherent imaging regime as in cELS presents some caveats [37]. In fluorescence microscopy, the fluorescent molecules which are typically a few nanometers in size emit independently and portray a linear mapping between the fluorophore concentration and image plane. But the partial coherent imaging nature of cELS implies that the sample plane concentration and image plane intensity obey a nonlinear relationship due to the interference term described in Eq. (5). This can lead to artificial sharpening and false localizations by the algorithm and hence, lead to artifacts in the reconstructed image. E.g., consider the scenario presented in case 1 above, where the particles are no longer resolvable as per Rayleigh’s criteria. The algorithm may then localize to the point of maximum intensity which lies in between the two particles, thus leading to a false localization. However, if the particles are resolvable as per the scenario presented in case 2 above, the algorithm can artificially sharpen the image and lead to a seemingly improved contrast and resolution. The nonlinear sharpening effect can also lead to masking of regions with lower scattering intensity.

## 4 Results and discussion

The following imaging results are presented to validate the theory developed above: (a) 60 nm polystyrene nanobeads, (b) weakly scattering nanosized biological specimens like liposomes and extracellular vesicles, (c) fixed and live biological cells, (d) 100 nm gold nanoparticles imaged using dark-field and cELS microscopy and (e) application of intensity fluctuation algorithms on 100 nm polystyrene nanobeads. (a–d) validate superior contrast imaging and (e) verifies the feasibility of applying intensity fluctuation algorithms in label-free mode. Details of experimental parameters are provided in Supplementary material Table 2.

### 4.1 Weakly scattering specimens

**Figure 3:**
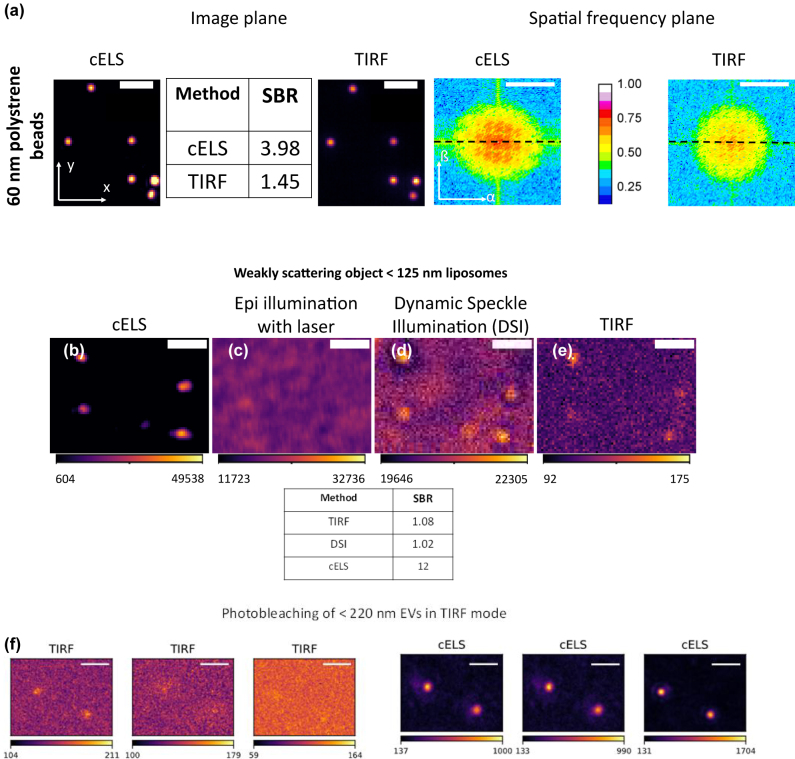
Experimental results of weakly scattering specimens. (a) cELS and TIRF images of 60 nm polystyrene beads. The signal to background ratio (SBR) is given in the table and the Fourier transform of the beads imaged using cELS and TIRF is given alongside. Scale bar 2 μm in the image plane (*x*–*y* plane) and 5 μm^−1^ in the spatial frequency plane (*α*–*ß* plane). The colorbar shows the intensity variation along the dotted lines in the Fourier plane. (b) Liposomes of <125 nm in size imaged using epi-illumination laser mode, (c) TIRF mode, (d) Dynamic Speckle Illumination mode and (e) cELS are compared. The colorbar shows the pixel values of the images. The corresponding SBR is given in the table below. Scale bar 2 μm. (f) cELS and TIRF images of <225 nm sized extracellular vesicles (EVs). The TIRF image shows photo-bleaching with time whereas cELS allows long-term imaging of the EVs. A larger field-of-view image of EVs is provided in Supplementary article, Figure S7. The colorbar shows the pixel values of the images. Scale bar 2 μm.

Firstly, 60 nm polystyrene nanobeads are imaged to compare the performance of TIRF and cELS. Figure 3(a) shows the images of 60 nm polystyrene beads acquired in cELS and TIRF mode and the two images are in good agreement. The signal to background ratio (SBR) is higher for the cELS image. The Fourier transform of the TIRF image shows that higher spatial frequencies get attenuated faster which is the case for incoherent imaging. On the other hand, cELS is a partially coherent imaging technique and therefore, the contrast does not drop significantly even for the higher spatial frequencies which is expected for coherent imaging. Thus, cELS supports superior contrast imaging of nano-sized structures that have predominantly high spatial frequencies. The difference in the Fourier spectrum between the coherent and incoherent imaging is further discussed in Supplementary material section S2 *.*

Next, we opted for samples that are both weakly scattering and are nanoscale in size, liposomes. The index contrast of liposomes with its surrounding is only about 0.04 [38] and the size of the liposomes used here is about 125 nm. This constitutes a weakly scattering specimen and hence, to detect these structures they are typically prepared including fluorescent molecules for fluorescence imaging. Due to their limited size, the fluorescence signal emitted by the structures is usually weak. But due to the use of high-index contrast waveguide material with a thin waveguide geometry (150 nm thick), the evanescent field intensity at the waveguide surface is high and decays rapidly, aiding in generating label-free images of such weakly scattering specimens with higher signal to background noise as opposed to TIRF. cELS image also shows the presence of a larger number of particles whereas the TIRF image of the same region of interest showed a fewer number of particles, see also Figure S6 of supplementary material. This could be attributed to bleaching out of the fluorescence or due to a very weak fluorescence signal. A similar behavior is also noted in Ref. [25].

In Figure 3(b)–(e), we compare the images of liposomes acquired using different light sources and in different modes. Here, both laser and pseudo-thermal light sources (PTS) are used in epi-illumination mode, and TIRF and cELS in the near-field illumination mode via a photonic-chip. A PTS is generated by passing a laser through a rotating diffuser to reduce the coherent noise [36] and such an illumination method is termed dynamic speckle illumination (DSI). As anticipated, the laser in the epi-fluorescence mode generates coherent noise that hinders label-free imaging of weakly scattering nano-object, Figure 3(b). The coherent noise, however, can be reduced using DSI. Even after addressing the coherent noise issue, the epi-configuration illumination mode generates a background signal comparable to that of the weakly scattered light from the object, consequently reducing the contrast of the images. In TIRF, fluorescence tagging, and near-field excitation helps reduce out-of-focus light to improve the image contrast. However, this method still suffers from photo-bleaching, labeling nonuniformity and background fluorescence signal arising from unspecific labeling that are inherent to fluorescence-based approaches. Contrary to all these approaches, cELS generates superior contrast imaging of liposomes in label-free mode, which is devoid of bleaching issues as well, as shown next.

Figure 3(f) shows the time-lapse imaging of another weakly scattering object, <225 nm extra-cellular vesicles (EVs). Photo-bleaching is a well-known problem in fluorescence microscopy and the bleaching of the fluorescence signal from EVs is depicted in the TIRF images. The fluorescent molecules bleach out over time in TIRF mode whereas cELS continued to generate high-contrast images of EVs even after photo-bleaching of its fluorescence. This demonstrates the time-lapse label-free imaging capability of cELS that would find application for imaging nano-sized biological structures like liposomes or EVs where the fluorescence signal will be limited. Also, cELS can be combined with image segmentation algorithms for estimating nano-particle density for different scattering intensities, as shown in Supplementary Figure S7.

**Figure 4:**
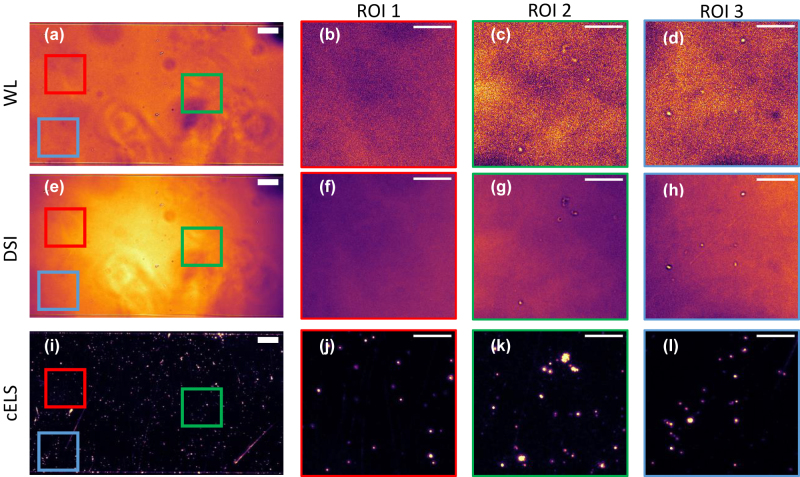
100 nm polystyrene beads imaged using 20×/0.45NA with white light (WL), dynamic speckle illumination (DSI) and cELS. (a, e, i) 100 nm beads imaged using WL, DSI and cELS respectively. Scale bar 50 μm. A few regions of interest, ROI 1–3, with aggregated and sparse beads are chosen within the FoV enclosed by red, green and blue boxes which are blown up and displayed. (b, c, d) WL images of 100 nm beads, (f, g, h) 100 nm beads imaged in DSI mode and (j, k, l) 100 nm beads imaged using cELS. Scale bar 20 μm in the blown-up regions.

Next, we demonstrate the competitive edge of cELS over incoherent epi-illumination methods. The decoupled illumination/detection paths of cELS configuration allow the use of a low magnification objective lens. This supports imaging of large areas without sacrificing the optical sectioning supported by the evanescent field. In Figure 4, 100 nm polystyrene beads are imaged in cELS and epi-illumination mode with both DSI and white light (WL) sources using a low magnification 20×/0.45N.A objective lens. The isolated nano-beads are not visible with DSI and WL. Only aggregated 100 nm beads are barely visible with DSI and white light sources. On the contrary, cELS provide high-contrast images even with 20×/0.45N.A. objective lens, thus enabling superior contrast imaging over large FoV. This is attributed to a multitude of factors like decoupled illumination/detection in dark-field mode, coherent scattering of multiple modes and use of high-index contrast waveguide material. The high effective index of the guided modes (*n*_eff_ = 1.75) scatters the dominant high spatial frequency components of the nano-sized samples. On the other hand, in epi-illumination mode, the illumination and detection schemes are coupled and both the light sources, i.e., spatially incoherent light (partially incoherent) for DSI and temporally incoherent light for WL fail to generate sufficient contrast. See Supplementary Figure S8 for scalable field of view imaging of 100 nm polystyrene beads in cELS mode using 25×/0.85NA and 60×/1.2NA MOs.

**Figure 5:**
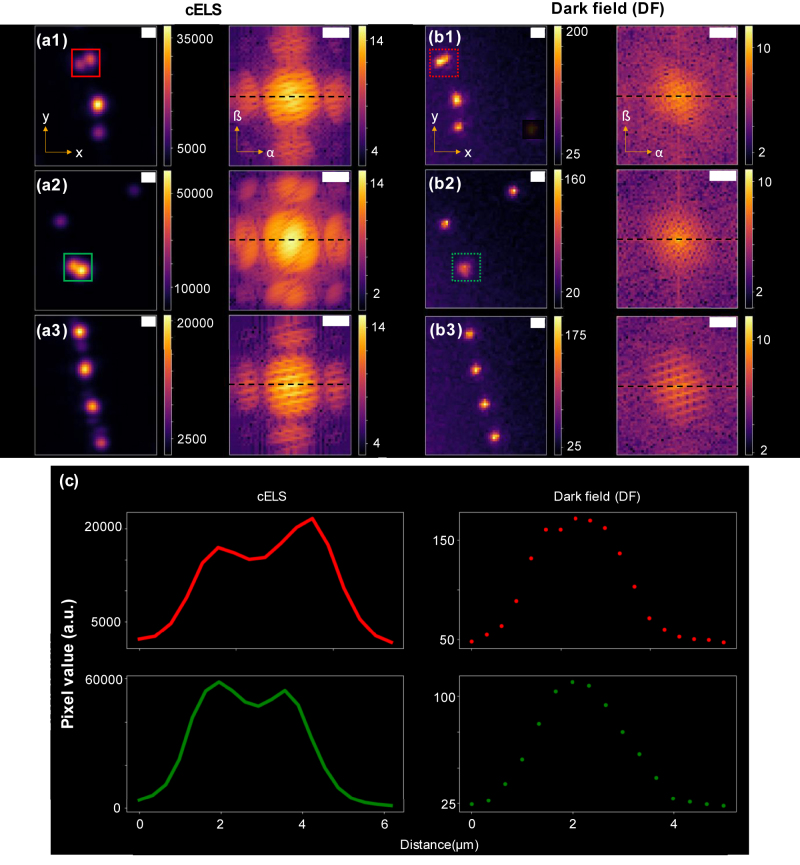
Comparison of 100 nm gold nanoparticles (GNP) imaged using cELS and dark-field (DF) microscopy. (a1)–(a3) Averaged image (*x*–*y* plane) of 100 nm GNP of three different regions of interest imaged in cELS mode and acquired using a 10X/0.25 NA MO. Their corresponding Fourier spectrum (*α*–*ß* plane) is shown alongside. (b1)–(b3) 100 nm beads imaged (*x*–*y* plane) using a DF microscope with 10×/0.3 NA and their corresponding Fourier spectrum (*α*–*ß* plane) is shown. The colorbars indicate the pixel values in the real image (*x*–*y* plane). In the Fourier images (*α*–*ß* plane) the colorbars indicate logarithm of the pixel values along the black dotted line. (c) Line plots of the cELS and DF images are shown. The line plots given by the red and green lines correspond to the boxes in (a1)–(a2) and are for the cELS images. The red and green dotted lines correspond to the dotted boxes in (b1)–(b2) and are for the DF images. To match the magnification between cELS and DF microscope, cELS images are bilinearly interpolated and displayed here. All the Fourier images are displayed on log scale for a better visualization. Scale bar 2 μm in real space and 500 mm^−1^ in Fourier space.

The resolution supported by cELS is *λ*_vac_/(*n*_eff_ + N.A.). While for other methods that use the same MO for illumination and collection, the resolution supported is given by *λ*_vac_/2N.A. As *n*_eff_ is independent of the imaging objective lens, even with a lower N.A. MO, cELS enables high-contrast and higher resolution imaging. To validate this point experimentally, a comparison between dark-field (DF) microscopy and cELS is demonstrated in Figure 5. For this experiment, 100 nm gold nanoparticles (GNP) are imaged in cELS mode using a 10X/0.25 NA MO and in DF mode using a 10X/0.30 NA MO. In cELS, the camera acquisition time is set at 1 ms and an image stack of 100 images is acquired. Three different regions of interest in the acquired image stack are shown in Figure 5(a1)–(a3). The same regions of interest are also imaged using DF microscopy. The exposure time of the DF microscope is set at 100 ms for a fair comparison between the two techniques and the images are given in Figure 5(b1)–(b3). Comparing Figure 5(a1) and (b1), the beads contained within the red box are resolvable as two distinct beads in cELS method, as shown by the line plot in Figure 5(c). A similar improvement in performance of cELS over DF is visible in the green boxes shown in Figure 5(a2) and (b2). The corresponding Fourier spectrum of the images is also provided alongside each of the images. The difference in the spectrum is attributed to cELS being a partially coherent imaging technique while DF microscopy uses an incoherent white light source for imaging. Also, due to the use of coherent laser and high effective index of the guided modes, the scattering signal in cELS images is almost two orders of magnitude higher than the corresponding DF images.

### 4.2 cELS for imaging cells

**Figure 6:**
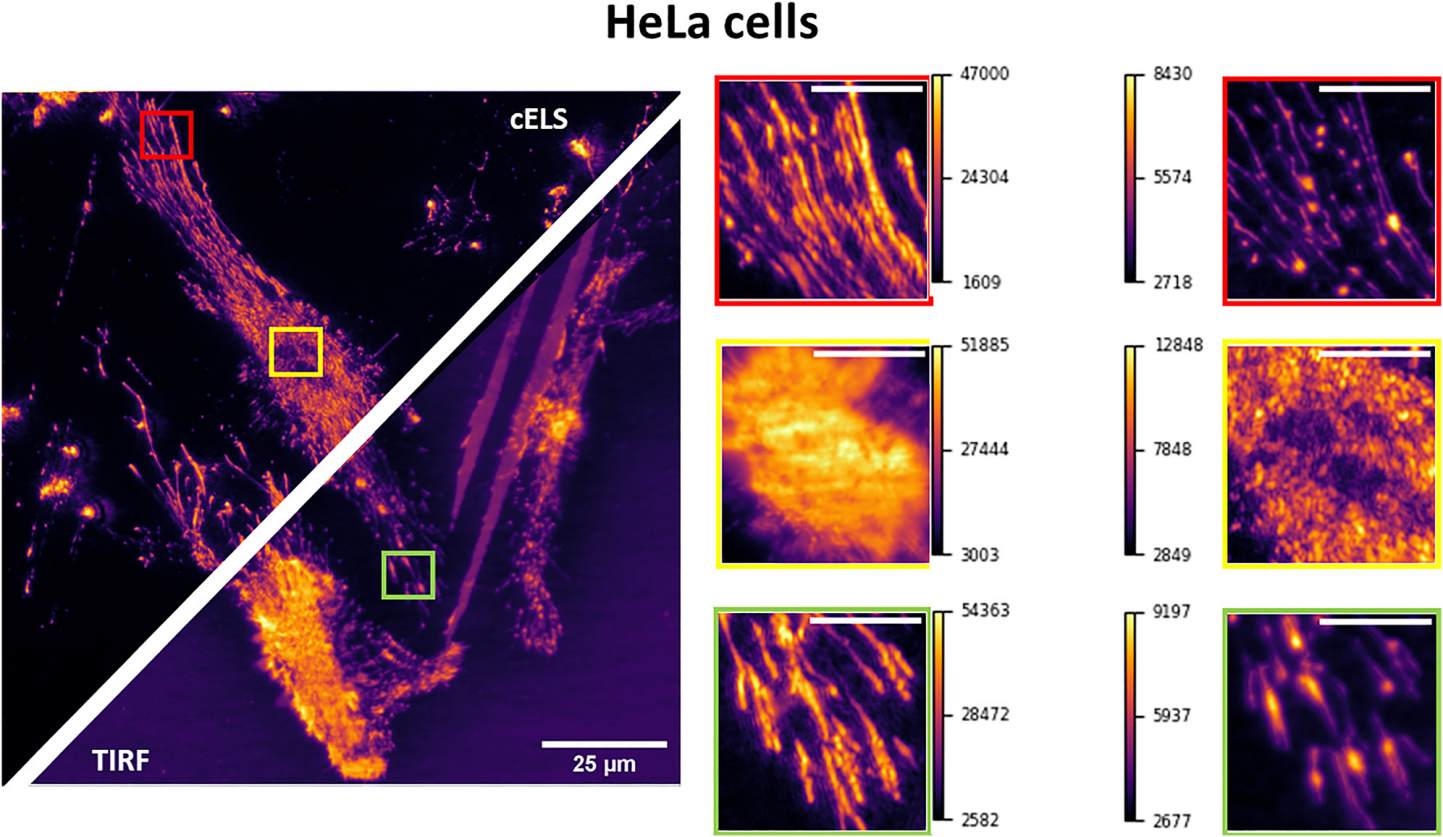
Comparison between cELS and TIRF images of Hela cells, scale bar 25 μm. Three different regions of interest enclosed by red, yellow, and green boxes are blown-up and provided alongside. The yellow box shows the nucleus region of the cell whereas the red and green boxes are the filaments, scale bar 8 μm. The color bars given alongside the magnified regions indicate the pixel values.

Here the compatibility of cELS for bioimaging is demonstrated and compared with fluorescence imaging. Figure 6 compares cELS and TIRF images of a fixed HeLa cell. cELS imaging was performed at 660 nm excitation and detection. For TIRF imaging, the actin filaments were labeled at 532 nm excitation and the Stoke shifted signal was detected using a 595/40 nm band-pass filter. Three different boxes in red, yellow, and green are blown-up and shown alongside for both cELS and TIRF. The yellow box contains the nucleus of the cell. Typically, the nucleus of the cell accommodates many fluorophores. As a result, the fluorescence intensity even in TIRF mode will be high. This can obscure some of the features as opposed to cELS. cELS image shows more features as the nucleus is situated slightly above the cell membrane and hence the evanescent field scattering will be less. Next for regions outside the nucleus, the TIRF image exhibited a reduced contrast for the filament like structures which could be attributed to weak fluorescence intensity, nonuniform, and unspecific labeling. Being a label-free method would enable cELS to perform long duration live cell imaging without worrying about photo-bleaching. In Figure S9 of the supplementary material, epifluorescence, TIRF and cELS images of the same region of interest presented here are given. The dynamics of a living HeLa cell acquired in cELS mode is provided as a supplementary movie.

**Video 1:** 

### 4.3 Sample illumination via four-arm crossing waveguide and application of fluorescence-based intensity-fluctuation algorithm to cELS

**Figure 7:**
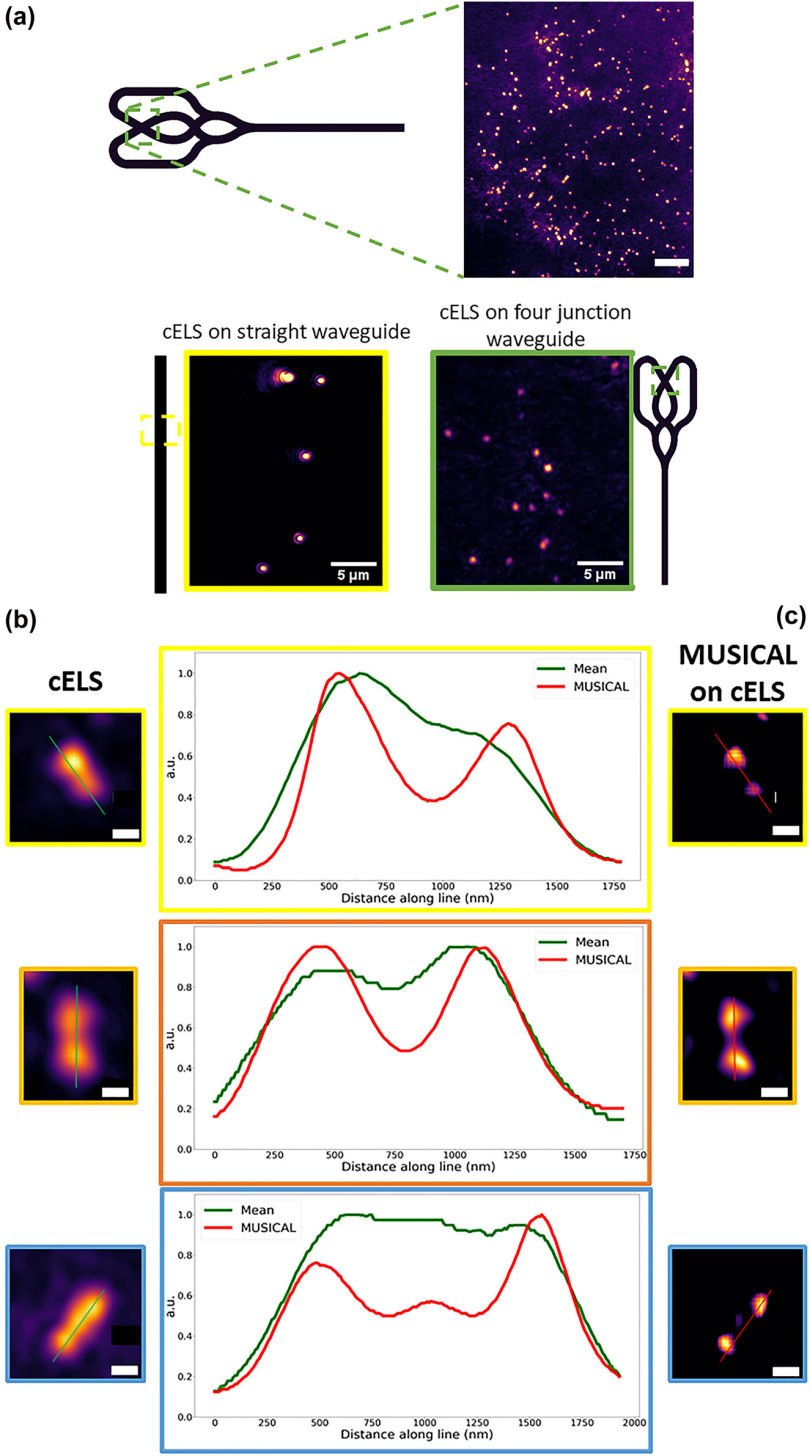
cELS on four arm junction waveguide and application of intensity fluctuation algorithm to cELS images. (a) Four arm junction waveguide used for the experiments. 100 nm polystyrene beads imaged in cELS mode using a four-arm crossing waveguide is given, scale bar 10 μm. The green dotted box in the waveguide shows the imaging region. The use of a four-arm crossing waveguide mitigates the coherent scattering noise as shown by the images of 100 nm polystyrene beads imaged using straight and four crossing waveguides. Scale bar 5 μm. (b) Three different regions of interest of 100 nm polystyrene beads imaged in cELS mode. (c) The corresponding MUSICAL reconstruction is shown. A stack of 100 images is given as input for MUSICAL. Three separate regions of interest in yellow, orange, and blue boxes in cELS and the corresponding MUSICAL reconstructions are shown side by side. The line profiles of the regions are given as well. Scale bar 500 nm.

A multi-moded straight waveguide supports modes predominantly along a straight line. For the waveguide geometry shown in Figure S3 of the supplementary section, the angle the modes described by Eq. (S3) of the supplementary material make with respect to the optic axis (*z*-axis) is given by *θ* = cos^−1^ *ß*/*kn*_1_ [39]. The difference in *θ* between the first mode $\left(ß=1.75\right)$ and say the twentieth mode $\left(ß=1.76\right)$ is only about 5 degrees. This fact of limited angular diversity between the modes can also be understood from Figure S2 in supplementary section. It is known that the period of interference fringes is inversely proportional to the angle between the modes. The argument of the cosine function in Eq. (5) depends on the angle between the two interfering beams. Since the angle between the modes is small, the Fourier peaks of the cosine function are also located close to the origin, which is what is seen experimentally as shown in Figure S2. As a result, the enhancement in resolution is not isotropic. To mitigate this issue, a four-arm crossing waveguide is proposed, shown in Figure 7(a). The imaging region is highlighted by the green dotted lines in Figure 7(a) where several modes from the four-arms interfere. By illuminating the sample from several azimuthal orientations, the illumination frequencies become isotropic [27, 40], [41], [42]. This concept is illustrated experimentally in Figure 7(a) where 100 nm polystyrene beads are imaged using straight and four-arm junction waveguide. The images acquired using straight waveguides show the presence of coherent noise, predominantly along the direction of propagation of light. This is mitigated when illuminating the sample from all azimuthal directions as shown by the images of 100 nm polystyrene beads acquired using a four-arm crossing waveguide. Using a four-arm crossing waveguide, though we have more illumination frequencies illuminating the sample as shown in Figure S2 of the supplementary text, it is still not isotropic. Resolution enhancement will be predominantly along the direction of propagation of the light. To achieve isotropic resolution enhancement, the photonic-chip geometry used for structured illumination microscopy as in Ref. [30] needs to be adopted for cELS.

Next, we investigate the effect of MUltiple SIgnal Classification ALgorithm (MUSICAL) on the cELS data stack. MUSICAL helps extract sub-diffraction limit sized features from diffraction-limited image stacks like other intensity-fluctuation based fluorescence algorithms like SOFI (Super-resolution Optical Fluctuation Imaging) [43], SRRF (Super-Resolution Radial Fluctuations) [44], ESI (Entropy based Super-resolution Imaging) [45], 3B (Bayesian analysis of Blinking and Bleaching) [46], SACD (Super-resolution with Auto-Correlation two-step Deconvolution) [47]. Via singular value decomposition, MUSICAL decomposes the diffraction-limited image stack into spatial patterns in the shape of eigen-vectors and eigen values. Then based on the user input, the algorithm splits the eigen-vectors into two disjoint subsets – signal and noise to compute the final MUSICAL image which contains sub-diffraction limit sized features. For a more detailed analysis on MUSICAL, the readers may refer to [28].

The average diffraction-limited image of 100 nm polystyrene beads acquired in cELS mode is shown in Figure 7(a). Three different regions of interest from this diffraction-limited image are blown up and shown in Figure 7(b). The corresponding MUSICAL reconstructions are shown alongside. MUSICAL helps resolve beads better than the cELS image as explained earlier. However, caution must be exercised as artifacts could be introduced due to false localizations and artificial sharpening. The corresponding line profiles, green for cELS and red for MUSICAL on cELS, help illustrate the concepts explained.

## 5 Conclusions

There already exists high-contrast label-free imaging techniques like waveguide-based techniques which use index-matched waveguides or/and incoherent light source for sample illumination [23–25], on-chip Fourier Ptychography using eight single moded waveguides for sample illumination [27], interferometric techniques that can achieve nanoscale sensitivity [48–55] etc. In this article we have demonstrated how a high-index contrast multi-moded waveguide can be used as a partially coherent light source for high-contrast imaging with enhanced resolution. We developed the theoretical framework and demonstrated experimental results of label-free super-contrast high-resolution optical microscopy method using a photonic-chip. The detection sensitivity of cELS depends on waveguide material impurities and surface roughness. However, silicon nitride based waveguides are complementary-metal-oxide-semiconductor (CMOS) compatible, which is a mature process. Therefore, we expect an improvement in detection sensitivity as has been demonstrated in Ref. [56].

It is important to mention that the mixing of high and low spatial frequency components at the image plane due to convolution between the object and illuminating field spectrum as explained earlier can lead to image distortions, see Supplementary section S5 for a more detailed discussion. And since the mixing of high frequency signals leads to the generation of moiré patterns which is finally collected by the microscope objective, sub-diffraction limit sized features of the sample will be enlarged more in the image plane [41, 42]. All these issues can distort the final image at the camera plane. However, owing to the nanoscale size of samples explored in this work (EVs, nanobead and liposomes), these issues are not significant.

Waveguide based imaging is an attractive imaging modality as has been demonstrated by the growing research in this field. Demonstrating label-free superior contrast and high-resolution imaging using waveguide-based imaging technology provides an attractive route to the field of label-free super-contrast high-resolution microscopy. Recently, an affordable waveguide-based 3D printed microscope has been used to image SARS-CoV-2 viroids [57]. Also, multi-modal imaging techniques by combining 3D structured illumination microscopy and ODT [58, 59], 3D quantitative phase imaging and SOFI [60] etc. have been demonstrated to provide complimentary information. Multi-modal imaging on chip of nano-sized viruses, exosomes, EVs and single-cell organisms such as microalgae and bacteria using cELS would be attractive applications, especially when combined with micro-fluidics [61]. We anticipate the results presented in this article will aid researchers in further developing the field of label-free super-contrast high-resolution microscopy.

## Supplementary Material

Supplementary Material Details
