# Erratum to: Multi-moded high-index contrast optical waveguide for super-contrast high-resolution label-free microscopy

**DOI:** 10.1515/nanoph-2022-0500

**Published:** 2022-09-06

**Authors:** Nikhil Jayakumar, Firehun T. Dullo, Vishesh Dubey, Azeem Ahmad, Florian Ströhl, Jennifer Cauzzo, Eduarda Mazagao Guerreiro, Omri Snir, Natasa Skalko-Basnet, Krishna Agarwal, Balpreet Singh Ahluwalia

**Affiliations:** Department of Physics and Technology, UiT The Arctic University of Norway, Tromsø 9037, Norway; Department of Microsystems and Nanotechnology, SINTEF Digital, Gaustadalleen 23C, 0373 Oslo, Norway; Department of Pharmacy, Faculty of Health Sciences, UiT The Arctic University of Norway, Tromsø 9037, Norway; Department of Clinical Medicine, UiT The Arctic University of Norway, Tromsø 9037, Norway; Department of Clinical Science, Intervention and Technology, Karolinska Insitute, 17177 Stockholm, Sweden

After the publication of this article, the authors found that the yellow boxes (nucleus of the cell) in Figure 6 of the main article, corresponding to cELS and TIRF modalities, got swapped and is hereby corrected as:

**Figure 6:**
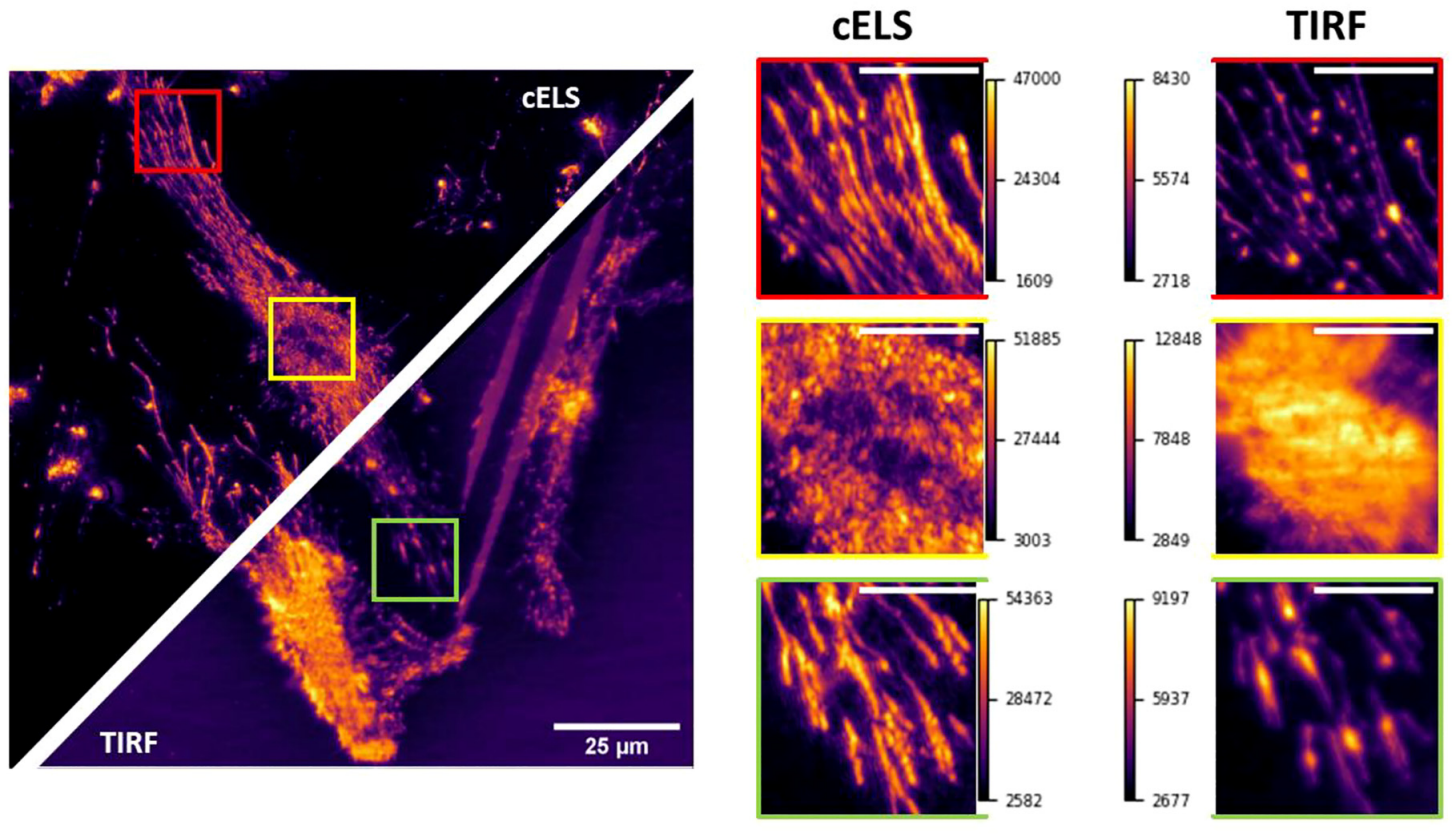
Comparison between cELS and TIRF images of Hela cells, scale bar 25 μm. Three different regions of interest enclosed by red, yellow, and green boxes are blown-up and provided alongside. The yellow box shows the nucleus region of the cell whereas the red and green boxes are the filaments, scale bar 8 μm. The color bars given alongside the magnified regions indicate the pixel values.

