# Supplementary material for: Multi-moded high-index contrast optical waveguide for super-contrast high-resolution label-free microscopy
Source: Nanophotonics. 2022 Jun 20;11(15):3421–36. doi: 10.1515/nanoph-2022-0100 (PMC10741054; doi:10.1515/nanoph-2022-0100)
Supplement: Supplementary file 3 — Supplementary Material Details [file j_nanoph-2022-0100_suppl.docx]

Multi-moded high-index contrast optical waveguide for super-contrast high-resolution label-free microscopy

Nikhil Jayakumar,^1,6^ firehun t dullo,^2^ vishesh dubey,^1^ azeem ahmad,^1^ Florian Ströhl, ^1^ jennifer cauzzo, ^3^ eduarda mazagao guerreiro, ^4^ omri snir, ^4^ natasa skalko-basnet, ^3^ krishna agarwal,^1^ and balpreet singh ahluwalia^1,5,7^

^1^ Department of Physics and Technology, UiT The Arctic University of Norway, Tromsø 9037, Norway

^2^ SINTEF Digital, Department of Microsystems and Nanotechnology, Gaustadalleen 23C, 0373 Oslo, Norway

^3^ Department of Pharmacy, Faculty of Health Sciences, UiT The Arctic University of Norway, Tromsø 9037, Norway

^4^ Department of Clinical Medicine, UiT The Arctic University of Norway, Tromsø 9037, Norway

^5^ Department of Clinical Science, Intervention and Technology, Karolinska Insitute, 17177 Stockholm, Sweden

^6^nik.jay.hil@gmail.com, ^7^balpreet.singh.ahluwalia@uit.no

This document supplements the work described in the main article. The concept of modes, the theory behind the formation of images in cELS and the application of cELS to imaging is provided here.

**S1. Optical modes in waveguides**

**FIG. S1. Schematic of a rectangular waveguide with a Gaussian mode profile being guided along its length.** The modes are field distributions that propagate keeping its transverse profile (x-y) intact. They merely accumulate a phase with distance ‘z’. A waveguide supports discrete and not a continuum set of modes, ψ_m_, and each mode is characterized by a discrete angle of propagation 𝜃_m_. Two-point particles, placed on the x-z plane, with a different refractive index scatters the evanescent field into the far-field.

Assume that the dielectric constant ‘$\epsilon$’ depends only on the transverse co-ordinate, x, and y. It means that along z-axis, the modes will accumulate only a phase as it propagates along the length of the waveguide. This will help reduce Maxwell’s equations to two independent sets of solutions called transverse electric (TE) and transverse magnetic modes. For TE modes the Helmholtz equation is given as

$(\frac{d^{2}}{dx^{2}}+\frac{d^{2}}{dy^{2}})E_{y}+{k_{0}}^{2}\epsilon\left( x,y \right)E_{y}=ß^{2}E_{y}$ (S1)

where E_y_ is the y-component of the electric field, k_0_ = ω/c is the free-space wave number and $ß$ is the propagation constant of the mode. For comparison, the two-dimensional time-independent Schrödinger equation is given, where $V\left( x,y \right)$ is the potential, $\psi_{(x,y)}$ is the wavefunction and E is the energy of the particle.

$\frac{{-h}^{2}}{2m}(\frac{d^{2}}{dx^{2}}+\frac{d^{2}}{dy^{2}})\psi_{(x,y)}+V\left( x,y \right)\psi\left( x,y \right)=E\psi\left( x,y \right)$ (S2)

For the waveguides used here the refractive index of the core n_1_ is greater than that of the substrates. Now comparing Eqn. (S1) and (S2), the dielectric constant is similar to the potential function. Or it means that light gets attracted to areas of higher dielectric constant, i.e., light gets attracted towards the core for the waveguides considered here. The solution of Eqn. (S1) can be found to be oscillatory in nature inside the core and the boundary conditions at the core-cladding interface impose decaying behavior for the evanescent fields. Now for a rectangular dielectric waveguide with dielectric constant $\epsilon\left( x,y \right)$, the general Helmholtz equation when solved yields the following solutions.

$$E_{x}\approx\cos\frac{m\pi x}{a}\sin\frac{n\pi y}{b}e^{-ißz}$$

$$E_{y}\approx\sin\frac{m\pi x}{a}\cos\frac{n\pi y}{b}e^{-ißz}$$

(S3)

$$H_{x}\approx\sin\frac{m\pi x}{a}\cos\frac{n\pi y}{b}e^{-ißz}$$

$$H_{y}\approx\cos\frac{m\pi x}{a}\sin\frac{n\pi y}{b}e^{-ißz}$$

**FIG. S2.** The multiple modes overlap to produce speckle patterns. (a) The use of straight waveguides limits the illumination spatial frequencies to regions around the vertical axis, ß_str_. This can be understood from the Fourier spectrum of the speckle pattern shown alongside. The blue and green triangles mark the cut-off frequency along the (α_str_-ß_str_) plane of the Fourier space for the straight waveguide. (b) To mitigate this issue and ensure isotropic sample illumination a four-arm crossing waveguide is proposed. Such an illumination geometry provides more illumination frequencies to the sample. This is demonstrated by the Fourier spectrum of the illumination frequencies shown alongside. The blue and green triangles mark the cut-off frequency along the (α_cross_-ß_cross_) plane of the Fourier space for the four-crossing waveguide. Scale bar 25 μm in real space images and 500 mm^-1^ in Fourier images. (c) The value of the Fourier spectrum along the blue and green triangles shown in (a) and (b) are plotted. Cut-off frequency along α_str_-ß_str_ of the straight waveguide and α_cross_-ß_cross_ of the four-arm crossing waveguide is marked.

The above equation constitutes the TE_mn_ modes where m = 0,1,2… and n = 0,1,2 etc. It implies that the modes are discrete and not continuous. Each of these modes can be also represented as a sum of two plane waves propagating at angles +𝜃 with respect to the z-axis as shown in Fig. S1. These modes propagate along the length of the waveguide through the generation of evanescent waves at the core-cladding interface. The physical understanding of the generation of evanescent waves can be as follows. The incident wave vector $\vec{k_{i}}=\alpha_{i}\hat{x}+ß_{i}\hat{y}+\gamma_{i}\hat{z}$ strikes the core-substrate interface as shown in Fig. S1. The dispersion relation for the wave vector in the core is given by ${\vec{k}_{i}}^{2}=\alpha_{i}^{2}+ß_{i}^{2}+\gamma_{i}^{2}$and for the cladding is ${\vec{k}_{cla}}^{2}=\alpha_{cla}^{2}+ß_{cla}^{2}+\gamma_{cla}^{2}$. The high index core squeezes the wave fronts at the interface. This implies that for phase matching at the core-cladding interface one of the wave vector components, $ß_{cla}$, of the cladding must become imaginary. This imaginary component of the wave vector leads to an exponential decay of the field away from the interface, i.e., along the y-axis in Fig. S1, and they are known as evanescent waves.

The discrete number of modes imply that there are discrete angles of propagation, and they can overlap in space and lead to speckles or multi-mode interference patterns as shown in Fig. S2. This makes the illumination profile non-uniform and speckle ridden. The Fourier spectrum of the illumination profile shows that in case of a straight waveguide, the illumination spatial frequencies are restricted along the k_y_ axis. As a result, isotropic resolution enhancement is not possible. Also, when intensity fluctuation based algorithms are applied on image stacks acquired using straight waveguides, the fluctuations in intensity will be predominantly along the vertical axis, thereby restricting the resolution enhancement only along the vertical axis.

**S2. Theory of image formation in cELS**

Consider two particles ‘1’ and ‘2’ placed on top of the waveguide surface as shown in Fig. S3. The input coherent laser light excites a few modes inside the waveguide. For clarity only two of these modes are depicted in Fig. S3. As mentioned in Ref. [1], any mode ‘m’ may be decomposed into a pair of plane waves propagating at angle +$\theta_{m}$ with respect to the z-axis. Therefore, two modes are represented in green (mode m1) and red color (mode m2) propagating inside the core. The tails of these modes extend into the cladding and interact with the particles located there. The particles are polarized as a result and radiate into the far-field which is detected by the camera.

**FIG. S3. Theory of cELS image formation.** Two modes of the waveguide m1 and m2 are shown in green and red color respectively. The modes are decomposed into a pair of plane waves propagating at discrete angles with respect to the optical axis along z. The evanescent tails of these guided modes interact with particles 1 and 2 placed on the surface and as a result, light is scattered into the far-field.

Neglecting the vectorial aspect of light, let the scalar field generated by the two particles be $U_{1}\left( \boldsymbol{r},t \right)=a_{1}\left( \boldsymbol{r} \right)e^{i\varphi_{1}(\boldsymbol{r},t)-i\omega t}$ and $U_{2}\left( \boldsymbol{r},t \right)=a_{2}\left( \boldsymbol{r} \right)e^{i\varphi_{2}\left( \boldsymbol{r},t \right)-i\omega t}$. For simplicity assume that the two particles are identical and radiate with the same amplitude distribution, i.e., $a_{1}\left( \boldsymbol{r} \right)=a_{2}\left( \boldsymbol{r} \right)=a\left( \boldsymbol{r} \right)$. The coherence lengths of waveguides have been experimentally calculated in Ref. [2] via the visibility of interference fringes. For silicon waveguides fabricated in standard CMOS foundry, the coherence lengths are found to be approximately 4.17 mm for rib and 1.6 mm for strip geometry [2] and it is about 27 m for phosphorous-doped core silica on silicon waveguides. The main source of fluctuations is attributed to variations in waveguide dimension and composition. These fluctuations manifest mainly in the phase of the light scattered by the particles and is accounted for by making the phase, $\varphi_{j}\left( \boldsymbol{r} \right)=\vec{k_{j}.}\vec{r.}$ , a function of time ‘t’, i.e., ${\varphi_{j}\left( \boldsymbol{r} \right)=\varphi}_{j}\left( \boldsymbol{r},t \right)$, where j=1,2 represent the two particles described here. The total electric field $U_{T}\left( \boldsymbol{r},t \right)$ reaching the camera will be

$U_{T}\left( \boldsymbol{r},t \right)={(U}_{1}\left( \boldsymbol{r},t \right)+U_{2}\left( \boldsymbol{r},t \right))⨷h(\boldsymbol{r})$ (S4)

where $h(\boldsymbol{r})$ is the coherent transfer function of the optical system, $⨷$ represents convolution operation and bold letters represent vectors. The spectrum of the total field reaching the camera may be computed as

$\tilde{U_{T}}\left( \boldsymbol{k},t \right)=\tilde{U_{1}}\left( \boldsymbol{k},t \right)\tilde{H}(\boldsymbol{k})+\tilde{U_{2}}\left( \boldsymbol{k},t \right)\tilde{H}(\boldsymbol{k})$ (S5)

where $\tilde{U_{j}}\left( \boldsymbol{k},t \right)$ is the Fourier transform of the amplitudes of the particles and $\tilde{H}(\boldsymbol{k})$ is the amplitude transfer function. The Fourier transform of the fields $U_{1}\left( \boldsymbol{r},t \right)$ and $U_{2}\left( \boldsymbol{r},t \right)$ may be computed as $\tilde{U_{1}}\left( \boldsymbol{k},t \right)=\tilde{A}\left( \boldsymbol{k} \right)⨷\delta\left( \boldsymbol{k-}\boldsymbol{k}_{\boldsymbol{1}} \right)\boldsymbol{=}\tilde{A}\left( \boldsymbol{k}_{\boldsymbol{1}} \right)$ and $\tilde{U_{2}}\left( \boldsymbol{k},t \right)=\tilde{A}\left( \boldsymbol{k} \right)⨷\delta\left( \boldsymbol{k-}\boldsymbol{k}_{\boldsymbol{2}} \right)\boldsymbol{=}\tilde{A}\left( \boldsymbol{k}_{\boldsymbol{2}} \right)$. Substituting this result into Eqn. (S5) we can see that the shifted object spectrum field is low passed by the coherent transfer function. Now the instantaneous intensity in real space can be computed from Eqn. (S4) as follows

$I_{T}\left( \boldsymbol{r},t \right)=\left[ {(U}_{1}\left( \boldsymbol{r},t \right)+U_{2}\left( \boldsymbol{r},t \right))⨷h(\boldsymbol{r}) \right].\left[ {{(U}_{1}}^{*}\left( \boldsymbol{r},t \right)+{U_{2}}^{*}\left( \boldsymbol{r},t \right))⨷h^{*}(\boldsymbol{r}) \right]$ (S6)

In this work, we scanned the input facet of the waveguide with a galvo to generate different sets of guided mode inside the waveguide. This generates speckle patterns that get added up incoherently during the integration time of the camera at the detector plane. This can be represented mathematically as

$\left\langle I_{T}\left( \boldsymbol{r},t \right) \right\rangle=I_{1}\left( \boldsymbol{r},t \right)+I_{2}\left( \boldsymbol{r},t \right)+\ldots=\sum_{n} I_{n}\left( \boldsymbol{r},t \right)$ (S7)

The final image generated by the camera is obtained as described by Eqn. (S7). To understand the Fourier transform of the cELS image generated, Eqn. (S6) may be Fourier transformed and then generalized for Eqn. (S7). Fourier transforming Eqn. (S6) gives

$\tilde{I}_{T}\left( \boldsymbol{k},t \right)=\left[ {(\tilde{U}}_{1}\left( \boldsymbol{k},t \right)+\tilde{U}_{2}\left( \boldsymbol{k},t \right)).\tilde{H}(\boldsymbol{k}) \right]⨷\left[ {{(\tilde{U}}_{1}}^{*}\left( \boldsymbol{k},t \right)+{\tilde{U}_{2}}^{*}\left( \boldsymbol{k},t \right)).\tilde{H}^{*}(\boldsymbol{k}) \right]$ (S8)

Substituting for $U_{j}\left( \boldsymbol{r},t \right)$ in Eqn. (S8), it can be further reduced to the following form.

$\tilde{I}_{T}\left( \boldsymbol{k},t \right)=\left[ \tilde{A}\left( \boldsymbol{k}_{\boldsymbol{1}} \right).\tilde{H}\left( \boldsymbol{k} \right)+\tilde{A}\left( \boldsymbol{k}_{\boldsymbol{2}} \right).\tilde{H}\left( \boldsymbol{k} \right) \right]⨷\left[ \tilde{A}^{*}\left( \boldsymbol{k}_{\boldsymbol{1}} \right).\tilde{H}^{*}\left( \boldsymbol{k} \right)+\tilde{A}^{*}\left( \boldsymbol{k}_{\boldsymbol{2}} \right).\tilde{H}^{*}\left( \boldsymbol{k} \right) \right]$ (S9)

where $\tilde{A}\left( \boldsymbol{k}_{\boldsymbol{1}} \right)$ and $\tilde{A}\left( \boldsymbol{k}_{\boldsymbol{2}} \right)$ represent the shifted Fourier spectrum of the two particles corresponding to the illumination fields exciting them, $\tilde{H}\left( \boldsymbol{k} \right)$ is the Fourier transform of the coherent transfer function and possess Hermitian symmetry, $\tilde{H}\left( \boldsymbol{-k} \right)=\tilde{H^{*}}\left( \boldsymbol{k} \right)$. The spectrum of the final image obtained will then be given by adding the spectra described by Eqn. (S9). For ease of notation let $\tilde{P}\left( \boldsymbol{k} \right)=\tilde{A}\left( \boldsymbol{k}_{\boldsymbol{1}} \right).\tilde{H}\left( \boldsymbol{k} \right)$ and $\tilde{Q}\left( \boldsymbol{k} \right)=\tilde{A}\left( \boldsymbol{k}_{\boldsymbol{2}} \right).\tilde{H}\left( \boldsymbol{k} \right)$. Then Eqn. (S9) can rewritten as

$\tilde{I}_{T}\left( \boldsymbol{k},t \right)=\tilde{P}\left( \boldsymbol{k} \right)⨷\tilde{P}^{*}\left( \boldsymbol{k} \right)+\tilde{Q}\left( \boldsymbol{k} \right)⨷\tilde{Q}^{*}\left( \boldsymbol{k} \right)+\tilde{P}\left( \boldsymbol{k} \right)⨷\tilde{Q}^{*}\left( \boldsymbol{k} \right)+\tilde{P}^{*}\left( \boldsymbol{k} \right)⨷\tilde{Q}\left( \boldsymbol{k} \right)$ (S10)

For incoherent illumination the cross-terms in the above-mentioned equation, $\tilde{P}\left( \boldsymbol{k} \right)⨷\tilde{Q}^{*}\left( \boldsymbol{k} \right)$ and $\tilde{P}^{*}\left( \boldsymbol{k} \right)⨷\tilde{Q}\left( \boldsymbol{k} \right)$, will tend to zero on time averaging and therefore, becomes linear in intensity. Thus, Eqn. (S10) represents the spectrum for coherent illumination. The spectrum of the final image will be the sum of many individual spectrums represented by Eqn. (S10) as explained earlier.

The following interesting cases may be noted while illuminating with waveguide modes. As per first order Born approximation, the incident and scattered waves can be assumed to have the same phase [3]. Therefore, the phase difference between the scattered waves off the two particles can be assumed to be dependent only on the illuminating field, the phase of which varies with the position of the particles. This can be understood as follows. Consider the situation where the two particles depicted as in Fig. (S3) are illuminated by the same mode. The two particles are assumed to be located at a separation of Rayleigh distance = 0.61λ/NA, where NA is the numerical aperture of the microscope objective and λ is the wavelength of detected light. In the experiments presented here, λ = 660 nm and NA = 1.2. Therefore, the two particles can be assumed to be located along the x-axis at (0, b/2, 0) and (0.508a, b/2, 0). In case of incoherent illumination, i.e., if fluorescent particles are used, they will be just resolved as per Rayleigh’s resolution criteria. However, in case of cELS the following cases may arise.

1. The two particles are illuminated by the same mode m1, see Fig. (S3). For example, consider illumination with TE_11_ mode (m=1, n=1). Substituting the particle’s location into Eqn. (S3) it can be seen that the phase difference between the scattered light reaching the detector will be approximately 1.6 radians. It implies that the interference term will not cause as much dip in intensity as observed in Rayleigh resolution limit, which corresponds to a phase difference of 𝛑 radians between the particles. It may be noted that the field equations given in Eqn. (S3) are the oscillatory solutions for the guided modes inside the core. For evanescent waves the same field distribution is obtained except for the fact that $\gamma_{i}$ becomes complex, i.e., exponentially decaying along the z-axis.
2. The two particles are illuminated by say TE_11_ and TE_21_ mode. Then the phase difference between the particles from Eqn. (S3), is found to be 𝛑 radians which corresponds to the particles being resolved as per Rayleigh’s criteria. Hence, a much deeper dip in intensity is observed than in the previous case.

Within the exposure time of the camera, the galvo would have oscillated several times back and forth. In the experiments presented here the galvo oscillation rate is set as a prime number at 1013 Hz. The exposure time of the camera was typically set to 30 ms. It means that the galvo would have oscillated approximately 30 times back and forth within 30 ms. The phase of the light varies at a rate order of magnitude higher than the exposure time of the camera or the oscillation rate of the galvo. At each position of the galvo, it will excite a set of modes. The particles scatter the light that interferes at the camera plane. The galvo then moves to the next position and again the particles scatter light with a different phase difference between them as explained earlier. The final image generated by the camera at the end of it’s integration time will be the sum of the different intensity distributions of the light scattered by particles. The phase difference between light scattered off by the particles due to multi-mode illumination can be assumed to have values ranging between –𝛑 and +𝛑 radians. Therefore, the final image formed at the camera plane can be considered a superposition of different speckle patterns with different phase differences, and thus resembling a random walk. This helps mitigate speckle noise as illustrated in [4].

**S3. Role of coherence in image formation**

The longitudinal coherence lengths of a light source may be deduced from Wiener-Khintchin theorem and is given by [5]:

$\frac{1}{L_{c}}\approx\frac{2\sin^{2} \theta/2}{\lambda_{0}}+\frac{\Delta\lambda}{{\lambda_{0}}^{2}}\cos^{2} \theta/2$ (S10)

where L_c_ is the longitudinal coherence length as a function of both angular and temporal spectrum, $\lambda_{0}$is the center wavelength of the temporal spectrum of a source with temporal bandwidth Δ𝛌, $l_{c}\approx\frac{{\lambda_{0}}^{2}}{\Delta\lambda}$ is the temporal coherence length of the light source and $\theta$ is width of angular spectrum. The longitudinal coherence length gives the length over which the photons remain correlated. A similar expression as in Eqn. (S10) is also obtained for transverse coherence lengths [5]. The coherence volume of the field may be defined as the product of the transversal and longitudinal coherence lengths. Only photons within the coherence volume can interfere to generate a sustained interference pattern.

For the experiments presented in this article, cELS imaging have been carried out using a diode laser, Cobalt Flamenco 660 nm laser. The bandwidth of this laser is < 1 GHz as mentioned in the company website. This implies a coherence time of ≈ 159 ns. The exposure time of the camera is 30 ms and therefore, a number of different speckle patterns as described earlier will get generated within the integration time of the camera. As a result, the degree of coherence is not unity and therefore, using this laser itself some mitigation in speckle noise is achieved. In contrast, if fluorescence imaging is performed, for example such as TIRF mentioned in this article, a larger number of speckle patterns will get generated within the integration time of the camera. It means the use of fluorescence will yield a highly incoherent illumination. For example, if the bandwidth of fluorescence emission is 100 nm, then the coherence time of the laser can be shown to ≈ 3 fs. It means that more that 10^6^ speckle patterns as compared to cELS imaging will get generated within the exposure time of the camera and therefore, more reduction in speckle noise is observed.

**S4. Waveguide fabrication and characterization**

The imaging platform is based on silicon nitride (Si_3_N_4_) waveguides on a silicon wafer, fabricated by low-pressure chemical vapor deposition and reactive-ion etching as detailed in [6]. Waveguide chips were produced at the Institute of Microelectronics Barcelona (IMB-CNM, Spain). Waveguide fabrication steps as follows: a 2µm thick silica layer was grown thermally on the silicon wafer, followed by deposition of silicon nitride layer via low-pressure chemical vapor deposition (LPCVD) at 800^◦^C. The waveguide structures are then created via photolithography and reactive ion etching (RIE) to produce the required 2-D waveguides.

**
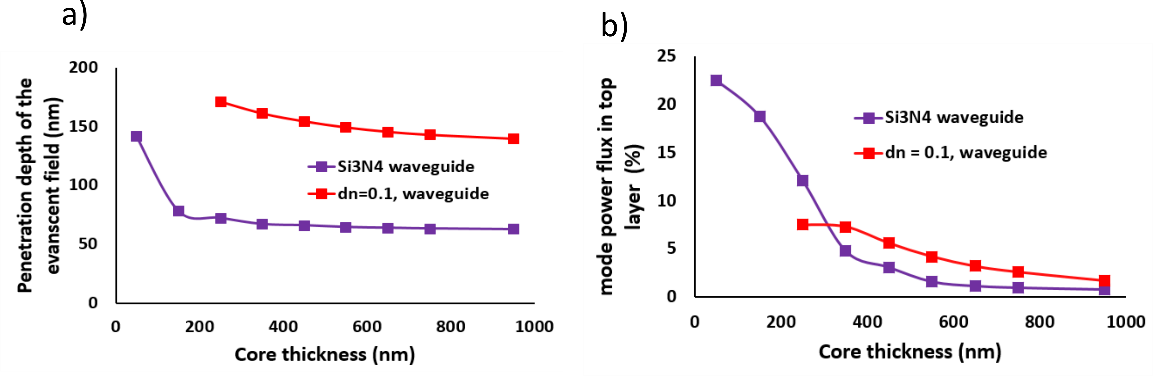
**

**FIG. S4.** The (a) penetration depth and (b) field intensity of a TE1 mode of Si_3_N_4_ high index core (Δn≈0.5) waveguide and an index matched waveguide (Δn≈0.1) are provided here.

| **Ref No. (Supp)** | **Title** | **Waveguide geometry and material** | **Excitation light for waveguide** | **Light registered by the camera** |
| --- | --- | --- | --- | --- |
| 7 | Optical Waveguide Microscopy | Planar geometry for the guiding structure. Two different core materials SiO_2_ and solid polyelectrolyte (polymer) used. | Coherent laser light | Coherent laser light |
| 8 | Waveguide Scattering Microscopy for Dark-Field Imaging and Spectroscopy of Photonic Nanostructures (WSM) | Dielectric slab waveguide (standard microscope slides or coverslips used for imaging.) | Incoherent white light | Incoherent white light |
| 9 | Fluorescent Nanowire Ring Illumination for Wide-Field Far-Field Subdiffraction Imaging | Film waveguide (SiO_2_, Al_2_O_3_ and TiO_2_) | Fluorescent light emitted from nanowire rings (incoherent broadband light) | Fluorescent light (incoherent broadband light) |
| 10 | Evanescent Light-Scattering Microscopy for Label-Free Interfacial Imaging: From Single Sub-100 nm Vesicles to Live Cells | Planar core (SiO_2_) with polymer cladding. | Coherent laser light | Coherent laser light |
| 11 | Super-condenser enables label-free nanoscopy | Si_3_N_4_ rib single moded waveguide | Coherent laser light | Coherent laser light |

Table 1: Comparison between different on-chip label-free microscopy works.

**S5. Liposomes preparation, characterization and labeling**

Labeled liposomes were formulated with a mass ratio 100:1 comprising soy phosphatidylcholine (SPC, Lipoid S100, main lipid ingredient kindly provided by Lipoid GmbH, Ludwigshafen, Germany) and 1-myristoyl-2-{6-[(7-nitro-2-1,3-benzoxadiazol-4-yl)amino]hexanoyl}-sn-glycero-3- phosphocholine (fluorescently labeled phospholipid, obtained from Avanti Polar Lipids, Alabaster, AL, USA). The film hydration method was used to prepare a multilamellar/multivesicular dispersion. A methanol solution of both ingredients was subjected to gentle solvent removal (Büchi Rotavapor, Büchi Labortechnik, Flawil, Switzerland) and the thin film obtained was rehydrated by hand shaking to the final concentration of 10 mg/mL of SPC and 0.1 mg/mL of labeled lipid. Stepwise extrusion through polycarbonate membranes (Nucleopore®) of 800, 400 and 200 nm sieving sizes was performed for efficient size reduction of the vesicles [12]. Hence, dynamic light scattering was utilized for conventional characterization (Malvern Zetasizer Nano – SZ, Malvern Oxford, UK), obtaining Gaussian distributions for size (121±30 nm, with polydispersity index of 0.19±0.01) and zeta-potential (-9.39±3.3 mV, as estimation of the surface charge).

**S6. EV preparation, characterization and labeling**

Isolation of platelet-derived extracellular vesicles

Blood (50 ml) was collected from healthy volunteers into non-glass Vacuette tubes (6ml Z with no additive, Greiner Bio-One, Austria). Acidic citrate dextrose (ACD, 39 mM citric acid, 75 mM sodium citrate, 135 mM [D]-glucose, pH 4.5) buffer was added immediately to the tubes to prevent clotting. Platelet rich plasma (PRP) was generated by centrifuging the blood at 140gx for 15 min with no brakes. PRP was transferred to fresh tubes and ACD and 2.83 µM Prostaglandin E1 (PGE1, Sigma) were added and centrifuged at 900 gx for 15 min to pellet the platelets. Supernatant was discarded and platelets were resuspended in Ca^2+^ free Tyrode-HEPES buffer (137 mM NaCl, 0.3 mM NaH_2_PO_4_, 3.5 mM HEPES, 5.5 mM [D]-glucose, pH 7.35) and washed twice by centrifuging at 900 gx for 15 min. Platelet washes were carried out in the presence of PGE1 at 2.83 µM to prevent their activation. Once washed, platelets were resuspended in Tyrode buffer (137 mM NaCl, 0.3 mM NaH_2_PO_4_, 3.5 mM HEPES, 5.5 mM [D]-glucose, 1 mM MgCl_2_, 2 mM CaCl_2_, 3 mM KCl, pH 7.35) to a final concentration of 250x10^6^ platelets/ml. Platelets were stimulated with 20 mM thrombin receptor activator peptide 6 (TRAP6, Sigma) for 15 min at 37 °C with and EDTA 20 mM was added to stop the reactions. To remove platelets, samples were centrifuged at 2,500 gx for 15 min and platelet-free supernatants were collected and stored at -80 °C until use. To purify EVs, supernatants were thawed and centrifuged at 20,000 gx for 30 min at 4 °C. EV pellets were resuspended in 0.1V of particle free Dulbeco’s Phosphate-buffered saline (DPBS, Sigma) and stained with 100 µl FITC-conjugated lactadherin (1.6 µM, Haematological Technologies). Importantly, to avoid sample contamination, FITC-conjugated lactadherin was pre-filtered using an Ultrafree MC-GV filter (Merck Millipore, Ireland). Extracellular vesicles were incubated with FITC- conjugated lactadherin for 15 min on ice, in the dark. Excess stain was washed by centrifuging at 20,000 gx for 30 min, 4 °C. Supernatants were discarded and extracellular vesicles were resuspended in 120 µl particle free DPBS, and filtered in a Ultrafree MC-GV filter at 5,000 gx for 30 sec. The study of extracellular vesicles was approved by the regional committee for medical and health research ethics (REC North) and donors have sighed an informed consent.

**S7. HeLa cell labeling**

HeLa cells were grown in the culture media prepared in minimum essential medium (MEM) supplemented with 10% fetal bovine serum and 1% penicillin/streptomycin. The cells were seeded into the PDMS well chambers located on waveguide chip and kept in a standard humidified incubator at 37°C with 5% CO2 overnight. For live-cell imaging, the culture medium was aspirated and replaced with pre-warmed Live Cell Imaging Solution (Invitrogen) before imaging. For actin labeling, HeLa cells were fixed for ~15 minutes using 4% paraformaldehyde in PBS. Cells were then washed in PBS followed by incubation with 0.1% Triton X-100 in PBS for 10 minutes. The cells were further washed 3 times in PBS for a few minutes each time. Cells were then incubated with Atto-565 phalloidin (1:50 for in PBS for 90 minutes). The quantum yield and extinction coefficient of Atto-565 phalloidin is 0.90 and 120000 cm^-1^M^-1^ respectively.

**FIG.S5. Comparison between epi-illumination, dark-field and photonic-chip based illumination.** Photonic-chip helps decouple the illumination and detection paths and caters to scalable field-of-view. The incident light is given in yellow and scattered light in blue.

**FIG. S6. TIRF, DSI and cELS images of < 125 nm liposomes.** The signal to background ratio is also given as inset in the figure. cELS shows the presence of larger number of scattering particles as opposed to the other methods of illumination. Scale bar 2 μm.

**FIG. S7. < 220 nm Extra-cellular vesicles imaged in cELS and TIRF mode.** (a) cELS and TIRF images of EV’s. Scale bar 30 μm. Magnified regions are also shown alongside. Scale bar 5 μm. (b) Histogram showing number of particles versus scattering intensity for the cELS image shown in (a).

**FIG. S8. Scalable field-of-view using cELS.** Decoupling of illumination and detection paths enable scalable field-of-view as illustrated here. A larger field-of-view (shown on left) of 100 nm polystyrene beads is imaged using a 25X/0.85 NA microscope objective initially and then for higher resolution, a smaller field-of-view is imaged using a 60X/1.2 NA microscope objective.

**FIG. S9. Comparison between epi-fluorescence, TIRF and cELS images of Hela cells with the same field-of-view.** Scale bar 25 μm.

| **Fig** | **Sample used** | **Experiment** | **Excitation λ** | **Detection λ** | **Exposure time** | **Filters used (LP –long pass, BP – band pass)** | **Additional comments** |
| --- | --- | --- | --- | --- | --- | --- | --- |
| 3(a) | 60 nm polystyrene beads | cELS | 660 nm | 660 nm | 30 ms | - |  |
|  |  | TIRF | 532 nm | 595 nm | 50 ms | 532 nm LP  595/40 nm BP |  |
| 3(b-e) | < 125 nm liposomes | Epi-illumination laser | 660 nm | 660 nm | 30 ms | - |  |
|  |  | TIRF | 488 nm | 520 nm | 200 ms | 488 nm LP  520/35 nm BP |  |
|  |  | Epi-illumination  spatially incoherent | 660 nm | 660 nm | 30 ms | - |  |
|  |  | cELS | 660 nm | 660 nm | 30 ms | - |  |
| 3(f) | < 225 nm extra-cellular vesicles | TIRF | 488 nm | 520 nm | 100 ms | 488 nm LP  520/35 nm BP |  |
|  |  | cELS | 660 nm | 660 nm | 30 ms | - |  |
| 4 | 100 nm polystyrene beads | 20X/0.45 NA (cELS, DSI, WL) | 660 nm | 660 nm | 30 ms | - |  |
| 5 | 100 nm gold nanoparticles | cELS | 660 nm | 660 nm | 1 ms |  | Stack of 100 images acquired in cELS mode for averaging. cELS performed using 10X/0.25NA and Dark-field using 10X/0.30 NA |
|  |  | Dark-field | White light | White light | 100 ms |  |  |
| 6 | Fixed Hela cells | cELS | 660 nm | 660 nm | 30 ms | - |  |
|  |  | TIRF | 532 nm | 595 nm | 50 ms | 532 nm LP  595/40 nm BP |  |
| 7(b-c) | 100 nm polystyrene beads | cELS/MUSICAL | 660 nm | 660 nm | 30 ms | - | Stack of 100 images acquired in cELS mode used as input for MUSICAL. Threshold value for MUSICAL set at 0.8 and subpixelation at 10. |

Table 2: Particulars of the experiments detailed in the main article.

**S8. Image distortions due to convolution between evanescent wave illumination and sample spectrum**

Fig.S10: Convolution between sample spectrum $\tilde{T}$(α) and illumination spectrum $\tilde{E}$(α), $\tilde{T}$(α)⨷$\tilde{E}$(α), shifts higher spatial frequencies of the sample into the pass-band of the microscope. Pass-band of the microscope is shown by the black dotted lines extending from -α_c_ to α_c_. The illumination spectrum consists of two delta functions at -α_eva_ and α_eva_ shown by the green and red arrows respectively.

Consider we have a one-dimensional sample as shown by the ground truth image in Fig. S11. A Fourier decomposition of the ground truth image gives rise to three spatial frequencies with different Fourier weights as shown, only positive frequencies are shown here for brevity. When such a sample is illuminated by an evanescent wave, say α_eva_=1 meter^-1^, the higher spatial frequencies of the sample convolve with the high spatial frequency of the illuminating field and get heterodyned. This is shown in Fig. S(11b) where all the three spatial frequencies of the ground truth image get shifted to a lower frequency. If these frequencies are not shifted back to their original positions in the Fourier space, this can lead to distortions or deviations from the ground truth image. This is shown in Fig. S(11c). However, this issue will not be severe when imaging nano-sized specimens such as liposomes or EVs which predominantly contain high spatial frequencies.

Fig. S11. (a) Ground truth image and its Fourier components. (b) Convolution of sample spectrum with an evanescent wave heterodynes the spatial frequencies of the sample spectrum, which can lead to distortions. The distorted image due to heterodyning and its Fourier components are shown. (c) Overlay of ground truth and the distorted images for visualization purpose.

References

1. GHATAK, AJOY AUTOR, et al. *An introduction to fiber optics*. Cambridge university press, 1998.
2. Yang, Yisu, et al. "Phase coherence length in silicon photonic platform." *Optics express* 23.13 (2015): 16890-16902.
3. Kong, Jin Au. "Theory of electromagnetic waves." *New York* (1975).
4. Goodman, Joseph W. *Statistical optics*. John Wiley & Sons, 2015.
5. Ryabukho, V. P., et al. "Wiener–Khintchin theorem for spatial coherence of optical wave field." *Journal of Optics* 15.2 (2013): 025405.
6. Prieto, Francisco, et al. "An integrated optical interferometric nanodevice based on silicon technology for biosensor applications." *Nanotechnology* 14.8 (2003): 907.
7. Hickel, Werner, and Wolfgang Knoll. "Optical waveguide microscopy." Applied physics letters 57.13 (1990): 1286-1288.
8. Hill, David J., et al. "Waveguide scattering microscopy for dark-field imaging and spectroscopy of photonic nanostructures." Acs Photonics 1.8 (2014): 725-731.
9. Liu, Xiaowei, et al. "Fluorescent nanowire ring illumination for wide-field far-field subdiffraction imaging." Physical review letters 118.7 (2017):076101.
10. Agnarsson, Bjorn, et al. "Evanescent light-scattering microscopy for label-free interfacial imaging: from single sub-100 nm vesicles to live cells." ACS nano 9.12 (2015): 11849-11862.
11. Ströhl, Florian, et al. "Super-condenser enables labelfree nanoscopy." Optics express 27.18 (2019): 25280-25292.
12. Cauzzo, Jennifer, et al. "Characterization of Liposomes Using Quantitative Phase Microscopy (QPM)." *Pharmaceutics* 13.5 (2021): 590.
